# Teleconsultation for eye health delivery

**Published:** 2022-06-07

**Authors:** Anthony Vipin Das

**Affiliations:** 1 Department of eyeSmart EMR & AEye: LV Prasad Eye Institute, Hyderabad, India.

**Teleconsultation has emerged as a significant component of eye health service delivery, especially given the challenges of accessing in-person health care services in the wake of the COVID-19 health care crisis.**

**Figure:**
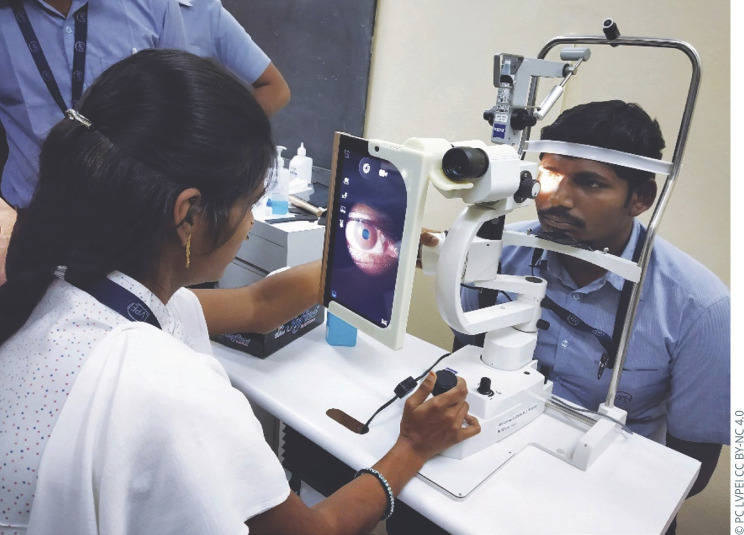
A slit lamp picture is captured at a vision centre in the primary eye care network and shared with the hospital, where an ophthalmologist views it and responds. **INDIA**

Teleconsultations – the use of communications technology to connect health professionals to each other and to patients – are important for providing health care to people in remote areas or to those unable to come to a secondary or teaching hospital, whether due to distance or the COVID-19 pandemic. Teleconsultation is not a new concept, but recent advances in technology solutions, the expansion of internet and cell phone connectivity, and the widespread use of smart devices have all contributed to its immense growth and popularity.

## What is teleconsultation?

Teleconsultation is a virtual medical consultation for diagnosis and/or treatment using information and communications technology (ICT) to bring health providers and patients together.

Teleconsultations can take place between the patient and physician, between physicians, between the physician and the primary care provider, between the patient and the primary care provider, or as three-way communication connecting the patient, the primary care provider, and the physician.

**Challenges and limitations of teleconsultations**

The following are some of the challenges and limitations associated with teleconsultations.

**Absence of physical examination.** If patients consult from home, e.g., via videoconferencing or telephone/cellphone, the primary eye care provider or ophthalmologist is unable to directly examine their eyes. Therefore, their advice will depend on the patient's description of their condition, or the quality of the video or photographs the patient can send. Likewise, when patients are seen at the primary level, the ophthalmologist has to depend on the available technology and the examination skills of the primary eye care provider, e.g., their ability to accurately measure intraocular pressure.

**Lack of adequate technology and infrastructure.** Internet connectivity in remote rural areas can be erratic, and the lack of hardware devices such as mobile phones or computers might hinder the patient's ability to perform a teleconsultation directly with the clinic. Staff members in primary care also need access to technology such as slit lamps with cameras or video cameras, and good quality internet connection so the images or videos can be transmitted electronically.

**Data security and regulatory barriers.** The exponential expansion of teleconsultation systems worldwide has created increased risks with respect to liability and legality. Data protection and data privacy are key issues that health care providers must be aware of, and comply with, to ensure patients’ privacy. It is recommended that digital health information systems are put in place to ensure patient data safety and continuity of care while using electronic medical records during teleconsultations and other interactions.^1^ Other regulatory matters to be aware of include country-specific licence and insurance requirements.

**Data accuracy and the potential for misdiagnosis.** Another key reason for setting up and maintaining digital health information systems is to ensure that patient data is accurate. This will help to avoid serious errors in the delivery of care.

Teleconsultations can be performed synchronously (i.e., in real-time, via video, audio, or text message interaction) or asynchronously (i.e., by transmitting or exchanging clinical information such as medical history, laboratory results, prescriptions, and so on).

## Benefits of teleconsultation

A well-designed teleconsultation service can:

- support diagnosis by providing timely access to the patient's medical information
- build consensus between different providers about the patient's care plan, thereby increasing the patient's trust in the health system
- contribute to the quality of the patient's overall experience of health care delivery.

## Teleconsultations in eye health care

Teleconsultations in eye health care (also known as teleophthalmology) can change the delivery of eye care from a centralised service to one which is patient-oriented and where decisions are made as close to the patient as possible. This reduces the need for an ophthalmologist to be present at every site.

In some countries, patients can attend consultations remotely via videoconferencing technology, which can be challenging due to connectivity issues or lack of access to technology. Another option is to connect a primary health care or eye care centre to a secondary or tertiary hospital staffed by ophthalmologists or specialist ophthalmologists, respectively. Teleconsultation allows non-specialist eye care providers working in remote areas – who are trained to use diagnostic equipment such as slit lamps or fundus cameras – to consult with expert colleagues so that anterior and posterior segment causes of avoidable blindness can be identified. Patients can then be referred to specialist centres to receive medical or surgical care.^2^^,^^3^ Teleophthalmology has been shown to be beneficial at the primary eye care level in screening for diabetic retinopathy,^4^ and it is a viable and cost-effective alternative to conventional eye care services in rural and remote areas.^5^

The philosophy of moving information instead of patients (for example, a technician screens for retinopathy of prematurity and sends images to be graded, instead of sending the parents and baby to a hospital) has benefits for the patient (by addressing socioeconomic and demographic barriers to accessing care) and for care providers (by optimising health care costs).

In summary, this **‘recognise, resolve, refer’** approach to teleophthalmology involves the following.

- **Recognise.** Timely **identification** of the common ocular conditions that cause visual impairment, such as pterygium, corneal ulcer, cataract, squint, and diabetic retinopathy, by the primary eyecare provider/ophthalmologist stationed at the base hospital.
- **Resolve.** Treatment advice provided by the ophthalmologist to the patient and primary eye care provider.
- **Refer.** Referral for more specialist medical or surgical care if needed.

**Figure:**
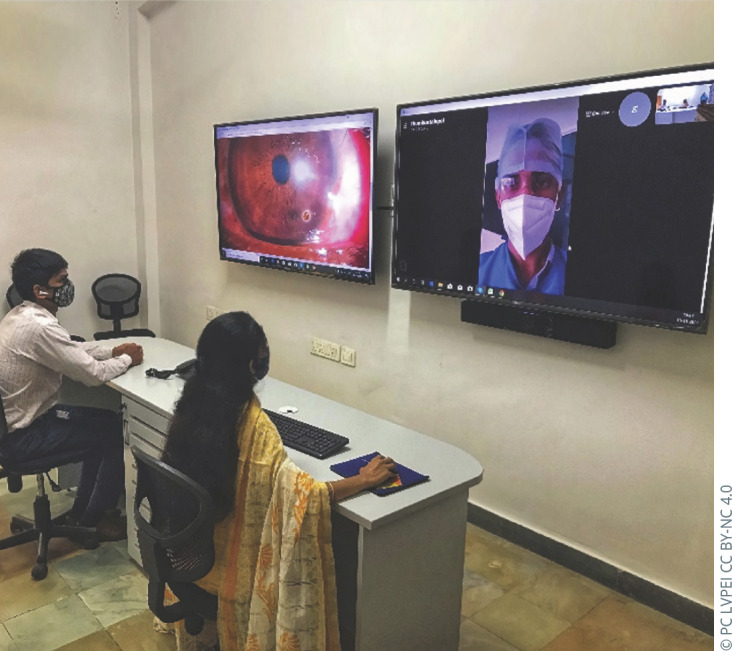
The command centre at the hospital receives the teleconsultation request from the primary eye care network and the ophthalmologists on duty respond. **INDIA**

**Teleconsultations in India during COVID-19**

In India, as the world over, the COVID-19 pandemic created many challenges in the area of health care, forcing health care providers to innovate and adapt quickly.

Forced to stay at home due to travel restrictions, patients and health providers turned to technology to communicate with one another, using apps or chat platforms such as Skype, Facetime, Zoom, Google Hangouts, Microsoft Teams, WhatsApp, Signal and Telegraph.

India's ministry of health and family welfare released telemedicine practice guidelines at the onset of the COVID-19 pandemic in 2020. The guidelines cover, among other points, definitions and applications of telemedicine, definitions of the registered medical professionals (RMPs) permitted to practice telemedicine, the technology used, patient consent, exchange of information for evaluating patients, and prescribing of medicines.^6^ Every RMP must complete a mandatory online course on telemedicine within three years of the notification of the course; in the interim, RMPs must follow the principles of the telemedicine practice guidelines.

Although there are still challenges to be overcome in health service delivery through telemedicine and teleconsultation, there is no doubt that there has been a significant increase in the use of teleconsultation services in India. The goal should be to harness the full potential of teleconsultations to benefit patients, enabling them to access care at locations closer to home and subsequently reduce the number of patients who need to be referred (for physical examination).
